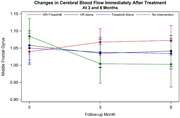# Virtual reality‐based cognitive‐motor training in middle‐aged adults at high Alzheimer’s disease risk improve cerebral blood flow in the frontal cortex: A randomized controlled trial

**DOI:** 10.1002/alz.092276

**Published:** 2025-01-09

**Authors:** Meir Plotnik, Abigail Livny, Maayan Harel, Glen M Doniger, Alex Bahar‐Fuchs, Yotam Bahat, Amihai Gottlieb, Ramit Ravona‐Springer, Gabi Zeilig, Michal S Beeri

**Affiliations:** ^1^ Tel Aviv University, Tel Aviv Israel; ^2^ Sheba Medical Center, Ramat Gan Israel; ^3^ Sackler Faculty of Medicine, Tel Aviv University, Tel Aviv Israel; ^4^ Sheba Medical Center, Tel Hashomer Israel; ^5^ The Australian National University, Canberra Australia; ^6^ Herbert and Jackeline Krieger Klein Alzheimer’s Research Center, Rutgers Biomedical and Health Sciences, Newark, NJ USA

## Abstract

**Background:**

Increasing prevalence of Alzheimer’s Disease (AD) and limited pharmacological intervention benefits to decelerate early neurodegeneration have prompted exploration of non‐pharmacological options. Recent studies indicate that combining cognitive‐motor training enhances outcomes.

**Methods:**

In a single‐blind, parallel‐group, randomized controlled trial of middle‐aged adults with a parental history of AD, the experimental group (N = 22) underwent training with newly developed “real‐world” intensive, progressive, virtual reality (VR) tasks, while walking on a treadmill. Tasks included challenges to sustained attention, selective attention, working memory, covert rule deduction, and planning, all in daily living contexts. An active control group without treadmill (N = 17) and another group walking on a treadmill while watching scientific documentaries (N = 16) were included. A passive control group received no training (N = 11). Training sessions were 45 minutes, twice a week, for twelve weeks. The primary neurobiological outcome, cerebral blood flow (CBF), in the superior (SFG), middle (MFG) and inferior (IFG) frontal gyri and middle temporal gyrus (MTG), was assessed using MRI arterial spin labeling at 12 weeks (i.e., post training) and at 6 months (3 months follow up). Linear mixed regression models, adjusting for age, sex, education and baseline cognition, assessed group differences.

**Results:**

Participants averaged 56.2 (SD = 5.78), were primarily female (70%) and had undergraduate education (mean = 16.4; SD = 3.12). The groups did not differ in any baseline characteristics. The interaction of group X time on CBF of the MFG was significant (p = 0.04), such that the experimental group had an increase in CBF at 3 months which then plateaued, there were no changes in CBF in the active control groups, and the passive control group had a decline in CBF (see **Figure**). Similar trends were found for the SFG and IFG (p for interaction 0.07 and 0.08, respectively). No differences were found between the groups in the MTG.

**Conclusion:**

The sustained increase in CBF, a major contributor to cognitive functioning, observed in the frontal cortex of the experimental group over three months, implies that our innovative VR intervention may beneficially affect cerebrovascular function, even in midlife. This trend underscores the potential effectiveness of our intervention in preserving healthy cognition among asymptomatic individuals at high AD risk.